# Hospital and Institutional News

**Published:** 1917-04-21

**Authors:** 


### HOSPITAL AND INSTITUTIONAL NEWS.

### THE OUTRAGES ON HOSPITAL SHIPS.

On April 13 the Admiralty announced the torpedoing on March 30 of the British hospital ship *Gloucester Castle*, an outrage which had already for some days been very widely bruited by rumour in London. Luckily there were no casualties, but this was not due to anything except the discipline and good conduct of the crew and staff, for there were four hundred wounded on board at the time. A few days later another British hospital ship, the *Salta*, struck a mine in the Channel during bad weather and sank: five doctors, nine nurses, and thirty-eight orderlies perished. As a reprisal for these deeds of savage barbarity, a German town has been heavily bombarded by French and British airmen. It is apparently thought that this is the punishment most likely to deter the authors of these crimes from repeating them; and it is to be hoped the event will prove that this view is correct. Probably the authorities have rejected the idea of carrying a couple of hundred German officers on the lowest deck of every hospital ship as merely likely to be seized as a pretext for greater barbarities to our unfortunate prisoners in Germany; and quite probably they are right.

### DEATH OF A LATE DIRECTOR-GENERAL OF THE ARMY MEDICAL SERVICE.

Sir William Taylor, late Director-General, died on April 10 at Windsor, aged seventy-four. He received his medical training at Glasgow University, and joined the Army Medical Staff—as it was then called—in 1864, when but twenty-one years of age. He was Principal Medical Officer in two campaigns—the Ashanti Expedition of 1895-96, and the Expedition to Khartum in 1898. His other war service included the Burmese Campaign, and the Chino-Japanese War, in which he was attached to the Japanese Army. From 1898 to 1901 he was Principal Medical Officer in India, and from 1901 to 1904 Director-General of the A.M.S. at home, retiring with the rank of K.C.B. and being succeeded by the present Director-General, Sir A. Keogh. Sir William Taylor was a Knight of Grace of the Order of St. John of Jerusalem in England, and an honorary LL.D. of Glasgow University.

### THE RANK OF CONSULTANTS IN THE R.A.M.C.

In a memorandum to Sir A. Keogh, Mr. Boyton, M.P. (who is member for the constituency which includes Harley Street and Cavendish Square, if we remember rightly), asked for the grant of the rank of major after a year's service to all consultants acting as surgeons, physicians, and specialists in Territorial hospitals. Sir Alfred Keogh refused the request in a reply the cogency of which seems to us unanswerable. This attempt to obtain for Harley Street specialists, already comfortably *embusqués*, as the French call it, in posts which allow them to live at home and conduct private practice, extra rank over the heads of those who bear the heat of the day at the Front was particularly ill-advised and ill-timed. Already these members of the profession enjoy the rank of captain immediately on their entry into the R.A.M.C., whereas all other doctors have to start as lieutenants. Even that measure of favouritism has been criticised a good deal in the profession at large as unjust and unnecessary. But at this particular juncture, when every doctor of military age is being relentlessly combed out all over the country, except the Harley Street dwellers, the move is even more likely to arouse resentment than it would at any other time. It is true that a good many of the younger men from Harley Street are under orders just now; but it is understood that the special privilege of doing only three months abroad, instead of a year like all other doctors, is being accorded to them. The reason for this special treatment is not obvious.

### TWO CRAFTSMEN AND QUEEN ALEXANDRA.

The needlework of soldiers has been referred to in these columns more than once, and its interest led Queen Alexandra to inspect the examples which have been produced by the soldiers under treatment at the Royal National Orthopædic Hospital in Great Portland Street. The work of two of the men pleased Queen Alexandra so much that she said she would like to purchase it. Its makers, however, excused themselves on the ground that they wished to show the work to their relations and to keep it in the family. Queen Alexandra, with a smile, respected their wishes, and withdrew her request. The craftman's pride in a new acquirement was aptly shown in this incident, and the work in question has thus gained a history which will doubtless be almost as much treasured by Queen Alexandra herself as by the men who were so genially harsh about their wishes.

### THE HUNS SURPASS THEMSELVES.

As horror is piled upon horror, and atrocity upon atrocity, one begins to wonder where the Boches will stop or how they get the ingenious ideas for outrage which have shocked the whole world. At present the record is held by their attempt to inoculate French non-combatants with tuberculosis, under the pretence of smallpox vaccination. In the nature of things this accusation is one very difficult to prove; indeed, without confession on the part of some conscience-stricken German, it will probably be impossible to prove it. But a distinguished Boston pathologist, Dr. Theodore Beebe, believes, as a result of his investigations among the French civilians in the evacuated territory of the St. Quentin district, that that is what the Germans have done. It is certainly significant that the Germans carried out an extensive campaign of vaccination a month or so before their retreat, when the Higher Command knew perfectly well that retreat was coming, and when every general, to say nothing of subordinates must have been preparing for withdrawal. It is also significant that only those who were inoculated, and no others, have developed tuberculosis since. Verily, the Hun is not fit to live

### MARRIAGE OF SIR W. J. THOMAS.

In our issue of November 11, 1916, the announcement was made of the engagement of Sir W. J. Thomas, of Ynishir, the Welsh coalowner and philanthropist, to Miss Maud Mary Cooper, the assistant matron of the King Edward VII. Hospital, Cardiff, which has been the recipient of so many benefactions from Sir William. Now we are pleased to be able to chronicle the marriage of this institutional pair, to whom we wish, in the names of all the hospital world, long life and happiness. The marriage was celebrated at St. Mary Abbot's Church, Kensington, by the Rev. Isaiah Roberts, vicar of St. Mark's, Newport. Among the wedding presents were gifts from public institutions with which Sir W. J. Thomas is associated, as well as from the miners of the Ynishir collieries. It is hardly necessary, perhaps, to remind our readers that the bridegroom has been a lavish and discriminating benefactor of the Welsh University Medical School in connection with the King Edward VII. Hospital; and that it was there he first met his wife —a romance of the kind more often met with in works of fiction than in real life.

### THE TWO PERILS TO SOLDIERS' PENSIONS.

We are glad to learn from the statement of Mr. G. N. Barnes, the Pensions Minister, that no inquiry as to the earnings of disabled men is in future to be made when their pensions are under consideration. The statement is important, since the public is growing impatient at the recent practice whereby a pledge, given with every pomp and circumstance, at a time when sacrifices are asked for, is later repudiated on the plea that circumstances alter cases. Any pledge can be conveniently broken on such a ground; and, in fact, the only value of a pledge is that it shall stand in future, in spite of some inconvenience to the authority which makes it. We uttered a warning note when it was first suggested that one convenient value of teaching a trade to permanently disabled men was that they might become thereby self-supporting, since this desirable result was coupled with the specious suggestion that such men would be too proud to accept a pension from the country they were not too proud to fight for. Sir A. G. Boscawen, Parliamentary Secretary to Mr. Barnes, emphasised the statement of his chief by declaring that " a man's pension was to be fixed solely in regard to his state of health or disability, and no account would be taken of his earnings." Steps must also be taken by Parliament to prevent employers reducing the wages of the disabled by the amount of their pensions, or at all.

### THE WALKER MEMORIAL AT PETERBOROUGH.

After a good deal of discussion the governors of Peterborough Infirmary have decided on the form which the memorial all desire to raise to the memory of the late Dr. Thomas James Walker, M.D., F.R.C.S., shall take. It has been a complicated question, since any addition to the present building has been vetoed on the ground that rebuilding must be undertaken before long. At the same time those responsible for the hospital see no early chance of raising the large sum of money which would be required for this purpose. A compromise has been decided on, which was due to the suggestion of Mr. J. H. Beeby—namely, to raise not less than £1,000, which would be temporarily invested for the endowment of a bed and a cot in the surgical ward, and when the time comes for rebuilding the entire institution, to devote the £1,000 towards an operating theatre dedicated in Dr. Walker's name. In this way the building fund is started, the memorial no longer delayed, and Dr. Walker's name at once associated with the present institution. It is an ingenious scheme, and if the committee never let the public forget that the £1,000 first asked for is only the nucleus of a larger sum they will have good cause to thank Mr. Beeby for having made it.

### A SPECIAL APPEAL FOR THE LIVERPOOL ROYAL SOUTHERN HOSPITAL.

At the annual meeting of this institution, held in February, the honorary treasurer, Mr. Lyon H. Maxwell, pointed out that the bank overdraft amounted to nearly £10,000, and suggested a special appeal for funds to liquidate it. Mr. Maxwell is now able to report a considerable measure of success in his appeal, which has been promoted mainly amongst shipowners and seafarers in general, because the Royal Southern is near the docks, and receives large numbers of accident cases from the shipping there. One old friend of the hospital has promised £1,000, provided that another £9,000 is raised before the end of April. Of this amount £3,780 has now been secured, and an urgent appeal is being circulated to the shipowners and dock-workers of Liverpool, and, in fact, to the citizens at large, to raise the remaining £5,220 before the end of the month, so as to secure the conditional offer already mentioned. The profits earned in the shipping industry since the war began, by both masters and men, have been so enormous that there should, one would think, be no difficulty in raising the money needed; and it will be a great feather in Mr. Maxwell's cap when the load of so heavy a debt is lifted from the institution as a result of his perseverance and energy.

### THE WORKS DEPARTMENT AT GUY'S.

The standard of the " up-keep " of the buildings and equipment at Guy's Hospital is a high one, and this fact must be attributed, firstly, to the wise policy of the executive, and, secondly, to the excellent works department which carries out their wishes, under the direction of the architect and surveyor, Mr. William J. Walford, whose annual report to the house committee has been published in the *Guy's Hospital Gazette*. The most important structural alterations in 1916 were an extension to the dining-hall in the Henrietta Raphael Nurses' Home, and three new clinical laboratories for the use of physicians and assistant surgeons.

Amongst the many surgical appliances and hospital furnishings made or repaired in the works department may be mentioned 992 crutches, 850 surgical instruments re-plated, 20 couches and settees re-upholstered, and 130 new window-blinds provided. The expenditure of the department for 1916 was £17,175. The stock of the several trades of the department was valued at £1,979 at the close of the year. The works department is responsible for the repairs and alterations of property of the hospital outside the hospital precincts and constructed last year an annexe to a workhouse in Southwark, thus securing an increase of £15 to the annual rent. The report contains particulars of the hospital fire insurances, which are for £325,550. The air-craft insurance premium amounted to £453.

### INFANT WELFARE WORK: THE NEW PHASE.

The Infant Welfare movement has reached what may be termed maturity, through the institutional development on which it is now entering. A sign of this is the meeting at the Mansion House on April 27, when Lord Rhondda and Mr. Balfour will speak on behalf of the Infant Welfare Appeal Committee of the Royal Free Hospital. The adjoining land, which has been given to the hospital, will enable its work to be extended, and the infant clinic, and the special ward reserved for babies who cannot be looked after in their own homes, can be developed by its means into something more than the consultative centre to which it has been limited at present. Since the Royal Free Hospital has already gained repute for its work in this direction, the developments ahead may serve as a model to London and other centres.

### SHOULD SALARIES BE PUBLISHED IN DETAIL?

Mr. Benwell Bird and the committee of the South Devon and East Cornwall Hospital have lately been criticised because the annual report of the institution gives the total and not the details of the salaries paid to the staff. It is suggested that this practice, which, by the way, is practically general, creates a bad impression on the public, which finds in it the appearance of something to hide. An analogy with the balance sheets of a commercial company is then drawn, and it is triumphantly concluded that the salaries of the staff are either adequate or inadequate, and that the subscribers have the right to know which! Probably the chairman and the committee do not care two straws whether the salaries are published in gross or in detail, but it never seems to have occurred to the critics that they may have been prevented by delicacy from putting forward the true reason—namely, the reluctance of the staff to have their incomes known. People are very secretive about their incomes; whether sensibly or not is a matter of opinion which does not alter the fact; and since the cherished secret has to be revealed when a claim for a rise in salary is made, there does not seem why, in the absence of special reasons, this secrecy should not be respected in the annual report.

### ACCIDENTS TO PATIENTS IN A DUNDEE INSTITUTION.

It seems to be clear from the statements and admissions lately made in respect of the East Poorhouse Hospital, Dundee, that there is no proper control in the institution, and that the remedies for this state of things, which everyone professes to regret, have been half-hearted and ineffective hitherto. When we learn that the medical officer, Dr. MacVicar, declared a few weeks ago that accidents to patients were becoming so frequent that he meant to send a report to the Local Government Board, we have heard enough to show lax and faulty administration. Unfortunately it was decided, apparently with Dr. MacVicar's consent, to appoint an extra charge nurse; but since the attendants whose duty it is to remain in the wards and assist the aged patients in and out of bed and across the polished floors are apparently generally "occupied with other duties elsewhere" at the times when the accidents occur, it is apparent that the staff is either too small or badly administered. Experience suggests that it is both. We therefore hope that the suggestion of getting more nurses will not be regarded as decisive, since numbers are no remedy for absence of control. It would be well if the President of the Local Government Board would take the hint already provided for him and send down an inspector to the East Poorhouse Hospital, Dundee, without waiting for the recurrence of an accident, that only a reform of administration and the removal of lax discipline can prevent.

### SCHOOL BUILDINGS CONVERTED TO HOSPITAL.

Field-Marshal Viscount French opened at Hull on Sunday last a new naval and military V.A.D. Hospital. In 1914 the Hull Education Committee were erecting on the Cottingham Road, at an estimated cost of £20,000, a girls' secondary school. The war, however, intervened when little beyond the brick and stone walls and the roof had been completed, and the work was thereupon suspended. The building was splendidly situated, set upon a terrace in an open position, with wide prospects of the surrounding country, and when the need for further hospital accommodation in the Humber Garrison area became urgent, the executive of the V.A.D. was struck with the idea that the place would make an ideal institution for the treatment of the sick and wounded. Lord Nunburnholme, Lord-Lieutenant of the East Riding, and Colonel Easton (County Director of the V.A.D.) were able without any difficulty to secure the use of the building from the Corporation, who also agreed to contribute £800 towards the cost of adapting it to the purposes of a hospital. An appeal for £18,000 met with an immediate and most generous response, and the work of conversion was pushed forward to completion with great rapidity. The hospital is equipped on the most modern lines. The fine assembly hall (74 feet by 40 feet), the gymnasium (55 feet by 30 feet), and the art-room (40 feet by 35 feet), have been converted into three great wards. In addition, twenty-five classrooms have been furnished as dormitories, and in all the beds total 450. The botany room, which leads to the conservatory, has been turned into the men's day-room, and the operating theatre, dispensary, and $x$-ray department are in other commodious and well-lighted apartments. The rooms available for the clothing and other stores and the baths were quite adequate as planned, and the only addition found necessary was the building of a large temporary kitchen on the playground site. This kitchen, 50 feet by 28 feet, is of timber, lined with fibrous material, and has an outer shell of corrugated iron. Its store-rooms are attached. The Commandant of the hospital is Lady Nunburnholme. The medical staff consists of one resident officer and eighteen local medical practitioners who have volunteered their services. The internal management is in the hands of an experienced matron, and a number of ladies have accepted appointments, without remuneration, as keepers of clothing and stores, and for clerical and other work.

### A FLOURISHING COUNTY NURSING ASSOCIATION.

The Duchess of Devonshire was re-elected president of the Derbyshire County Nursing Association at the annual meeting the other day, when reference was made to the proved wisdom of transferring the headquarters of the Association from Derby to Chesterfield. Not only has the organisation been brought into closer touch with a populous district, but the local Association, instead of raising with difficulty an annual income of £60, has obtained £400, and is engaging three additional nurses. The report of the Executive Committee states that there are sixty Associations in the county, and that seventy-two nurses are employed. There are prospects of several new branches being formed in the Chesterfield district. Owing to the shortage, considerable difficulty has been experienced in supplying relief nurses, and the executive warns the Association that if the work is to be maintained more nurses will have to be trained. Three pupils completed their training during the year and are now at work. Mr. E. Miller Mundy, of Shipley Hall, who presided, expressed the opinion that after the war it would not be difficult to procure funds for a vigorous nursing campaign.

### WOMEN DOCTORS FARM FOR DISABLED SOLDIERS.

Two women doctors, Dr. Annie McCall and Dr. M. Smith, are interesting themselves in the management of a co-operative farm for the employment of severely disabled sailors and soldiers. The farm is situated at Sutton Valence, about six miles from Maidstone, and is sixty-five acres in extent. It is hoped to build about thirty cottages, each of which will be the home of a disabled man and his family. As there is already a village near, and also motor-'bus communication with Maidstone three times a day, the men would not feel themselves cut off from social life, and there would be the ordinary educational facilities for their children. The farm is 400 feet above sea-level, so the air is pure and bracing. The president of the society which is developing the scheme is Sir Frederick Milner, who has already done so much for soldiers and whose interest in them is as deep as it is unselfish; while among the executive committee there are, besides the medical women we have mentioned, Miss Binnie Clark, the official organiser of "Women on the Land" for Yorkshire and Lincoln, and Miss Helen Colt, the well-known practical gardener of Bedford College. The hon. sec., Miss Violet Bertram, who has experience of work on the land not only in this country but in Canada and on the Continent, will reside permanently on the farm as unpaid managing director. Disabled soldiers, many of whom have had little or no previous experience of land work, will require personal help, guidance, and encouragement; but given these they will probably regain health and the self-respect that comes from useful work, and as the colony will not be large it should escape the taint of institutionalism.

### A FINE ACHIEVEMENT.

To close with a balance in hand on two successive years during wartime is a rare achievement, but it has been accomplished by the Lincoln County Hospital. It began last year with £60 to its credit, and begins the new financial year with £215. These figures, it should be added, were brought forward at the recent quarterly meeting, and therefore it would perhaps be hasty to suppose that each quarter is equally successful. Perhaps hardly less noteworthy is a recent gift of 30 cwt. of seed potatoes from Mr. W. M. Epton, of Croft, who has deservedly been made a life governor in consequence.

### THIS WEEK'S DRUG MARKET.

The week has witnessed an unusually small number of price changes of importance, and on the whole business has been quiet. Senega root is dearer. Colchicum corm is quoted at a very high price. Acetanilide tends upwards in price, but there is little change in the position of synthetic drugs generally. Barbitone, however, does not seem to be quite so scarce, and is available at a little below the price recently quoted. Tannic acid is again dearer. Quinine is unchanged. The continued severe weather is having a very unfavourable influence on English herbs, and both lavender and rosemary have been badly affected. Belladonna is also suffering, the roots in some places having rotted as a result of the saturated state of the soil. The henbane seeds ought to have been in the ground three or four weeks ago, but the excessive rains have hindered the sowing. So far as can be foreseen, America's entry into the war is not likely to have a great effect on the drug market; the United States has for a long time been sending us as large a supply of drugs as possible, and it is at least probable that the diversion of labour from the drug industries to the manufacture of munitions may result in some curtailment of the output of drugs in America. In view, however, of the promise of more ships for Anglo-American trade it is possible that in due course American drugs will be arriving with more regularity.

